# SARS-CoV-2 Vaccine Responses in Individuals with Antibody Deficiency: Findings from the COV-AD Study

**DOI:** 10.1007/s10875-022-01231-7

**Published:** 2022-04-14

**Authors:** Adrian M. Shields, Sian E. Faustini, Harriet J. Hill, Saly Al-Taei, Chloe Tanner, Fiona Ashford, Sarita Workman, Fernando Moreira, Nisha Verma, Hollie Wagg, Gail Heritage, Naomi Campton, Zania Stamataki, Paul Klenerman, James E. D. Thaventhiran, Sarah Goddard, Sarah Johnston, Aarnoud Huissoon, Claire Bethune, Suzanne Elcombe, David M. Lowe, Smita Y. Patel, Sinisa Savic, Siobhan O. Burns, Alex G. Richter, on behalf of the COV-AD consortium, Zahra Ahmed, Hollie Bancroft, Michelle Bates, Hayley Clifford, Georgina Davis, Joanne Dasgin, Mohammad Dinally, Fatima Dhalla, Elena Efstathiou, Shuayb Elkhalifa, Mark Gompels, Dan Hartland, Madeeha Hoque, Emily Heritage, Deborah Hughes, Ann Ivory, Rashmi Jain, Sinead Kelly, Theresa McCarthy, Christopher McGee, Daniel Mullan, Hadeil Morsi, Eileen O’Grady, Shannon Page, Nicholas Peters, Timothy Plant, Archana Shajidevadas, Malgorzata Slowinsksa, Zehra Suleiman, Neil Townsend, Charlotte Trinham, Stuart Wareham, Sinead Walder

**Affiliations:** 1 Clinical Immunology Service, Institute of Immunology and Immunotherapy, University of Birmingham, Birmingham, UK; 2 University Hospitals Birmingham NHS Foundation Trust, Birmingham, UK; 3 Institute of Immunology and Immunotherapy, University of Birmingham, Birmingham, UK; 4 Department of Immunology, Royal Free London NHS Foundation Trust, London, UK; 5 Institute of Translational Medicine, University of Birmingham, Birmingham, UK; 6 Nuffield Department of Medicine, University of Oxford, Oxford, UK; 7 Medical Research Council Toxicology Unit, University of Cambridge, Gleeson Building, Tennis Court Road, Cambridge, CB2 1QW UK; 8 Department of Clinical Immunology, University Hospitals North Midlands, Stoke-on-Trent, UK; 9 Department of Clinical Immunology, North Bristol NHS Trust, Bristol, UK; 10 Department of Allergy and Clinical Immunology, University Hospitals Plymouth NHS Trust, Plymouth, UK; 11 Department of Allergy and Clinical Immunology, Newcastle Upon Tyne Hospitals NHS Foundation Trust, Newcastle, UK; 12 Institute of Immunity and Transplantation, University College London, London, UK; 13 NIHR BRC Oxford Biomedical Centre, University of Oxford, Oxford, UK; 14 Department of Allergy and Clinical Immunology, Leeds Teaching Hospitals NHS Trust, Leeds, UK

**Keywords:** COVID-19, CVID, Inborn errors of immunity, Primary immunodeficiency, Secondary immunodeficiency, Vaccination, SARS-CoV-2

## Abstract

**Background:**

Vaccination prevents severe morbidity and mortality from COVID-19 in the general population. The immunogenicity and efficacy of SARS-CoV-2 vaccines in patients with antibody deficiency is poorly understood.

**Objectives:**

COVID-19 in patients with antibody deficiency (COV-AD) is a multi-site UK study that aims to determine the immune response to SARS-CoV-2 infection and vaccination in patients with primary or secondary antibody deficiency, a population that suffers from severe and recurrent infection and does not respond well to vaccination.

**Methods:**

Individuals on immunoglobulin replacement therapy or with an IgG less than 4 g/L receiving antibiotic prophylaxis were recruited from April 2021. Serological and cellular responses were determined using ELISA, live-virus neutralisation and interferon gamma release assays. SARS-CoV-2 infection and clearance were determined by PCR from serial nasopharyngeal swabs.

**Results:**

A total of 5.6% (*n* = 320) of the cohort reported prior SARS-CoV-2 infection, but only 0.3% remained PCR positive on study entry. Seropositivity, following two doses of SARS-CoV-2 vaccination, was 54.8% (*n* = 168) compared with 100% of healthy controls (*n* = 205). The magnitude of the antibody response and its neutralising capacity were both significantly reduced compared to controls. Participants vaccinated with the Pfizer/BioNTech vaccine were more likely to be seropositive (65.7% vs. 48.0%, *p* = 0.03) and have higher antibody levels compared with the AstraZeneca vaccine (IgGAM ratio 3.73 vs. 2.39, *p* = 0.0003). T cell responses post vaccination was demonstrable in 46.2% of participants and were associated with better antibody responses but there was no difference between the two vaccines. Eleven vaccine-breakthrough infections have occurred to date, 10 of them in recipients of the AstraZeneca vaccine.

**Conclusion:**

SARS-CoV-2 vaccines demonstrate reduced immunogenicity in patients with antibody deficiency with evidence of vaccine breakthrough infection.

**Supplementary Information:**

The online version contains supplementary material available at 10.1007/s10875-022-01231-7.

## Introduction

The immunological correlates of protection against SARS-CoV-2 infection and severe COVID-19 are not yet known. The passive acquisition [1] or development of anti-SARS-CoV-2 spike glycoprotein antibodies following infection [2–4] confers significant protection against future disease and, in some cases, facilitates viral clearance in individuals that fail to mount effective immune responses following infection [5–9].

Vaccination against SARS-CoV-2 is the most effective public health intervention to prevent severe morbidity and mortality from COVID-19 in the general population [10–12]. A meta-analysis of vaccine efficacy studies has suggested that neutralising antibody levels are strongly associated with protection from symptomatic infection [13]. However, it is well recognised that patients with immunodeficiency may not respond optimally to vaccination. To date, SARS-CoV-2 vaccine immunogenicity and efficacy has not been comprehensively studied in individuals with primary and secondary immunodeficiency; preliminary studies suggest seropositivity rates following vaccination vary between 20.0 and 83.0% [14–18]. Given the significantly increased risk of morbidity and mortality from COVID-19 that these patients face [19, 20], understanding the immunogenicity and efficacy of vaccines in this population is of critical importance.

COVID-19 in patients with antibody deficiency (COV-AD) is a multi-site UK study that aims to: (i) determine the prevalence of asymptomatic and symptomatic SARS-CoV-2 infection in patients with primary and secondary antibody deficiency, (ii) determine how frequently SARS-CoV-2 viral persistence occurs in patients with primary and secondary antibody deficiency and (iii) characterise the immune response of these patients following SARS-CoV-2 infection and vaccination. This manuscript presents an interim analysis of 320 participants in the COV-AD study to describe responses to the primary course of vaccination and the risk of vaccine breakthrough and viral persistence.

## Methods

### Patient Eligibility and Recruitment

From March 2021, patients with primary or secondary antibody deficiency were recruited from the following immunology centres across the UK: University Hospitals Birmingham NHS Foundation Trust, Royal Free London NHS Foundation Trust, North Bristol NHS Trust, Oxford University Hospitals NHS Foundation Trust, Leeds Teaching Hospitals NHS Trust, University Hospitals North Midlands NHS Trust, University Hospitals Plymouth NHS Trust, Newcastle Upon Tune Hospitals NHS Foundation Trust.

Patients were eligible for the study entry if (i) they were over 18 years of age and (ii) they were receiving immunoglobulin replacement therapy or they had a serum IgG concentration less than 4 g/L and were receiving regular antibiotic prophylaxis to prevent infections. Participants’ underlying immunological diagnosis was made according the European Society of Immunodeficiency Clinical Working Party criteria. In this manuscript, “other primary antibody deficiency” has been used to encompassing individuals who do not fulfil the diagnostic criteria for CVID, XLA or any monogenic immunodeficiency but are still believed to have a primary humoral immunodeficiency.

At study entry, meta-data including demographics, immunological diagnosis and immunological parameters (e.g. baseline IgG concentration, trough IgG concentration, lymphocyte enumeration and whether an individual had previously tested positive for SARS-CoV-2 by PCR) were documented. All participants submitted a postal nasopharyngeal swab to determine SARS-CoV-2 status by PCR as previously described [21]. Individuals with a positive SARS-CoV-2 PCR were sent follow-up swabs at two-weekly intervals until a negative swab was returned. Results of routine clinical swabs were also documented as part of this study.

Study participants were then followed longitudinally through the UK routine SARS-CoV-2 vaccination schedule. Participants received two doses of either the AstraZeneca ChAdOx1 nCoV-19 (Vaxzevria) or the Pfizer BioNTech 162b2 (Tozinameran) vaccine according to the extended vaccine schedule mandated by the UK Chief Medical Officers (https://www.gov.uk/government/publications/prioritising-the-first-covid-19-vaccine-dose-jcvi-statement/optimising-the-covid-19-vaccination-programme-for-maximum-short-term-impact).

A cohort of 205 healthy control participants was recruited from the COVID-19 convalescent (COCO) study undertaken at University Hospitals Birmingham NHS Foundation Trust. These participants were otherwise healthy health care workers (median age 44 years, (range 22–66 years), 28% male), vaccinated with two doses of Pfizer BioNTech 162b2 on the extended UK dosing schedule and sampled 1–2 month after vaccination.

Participants were sampled, whenever possible, prior to their second vaccine dose and between 1 and 2 months following their second vaccine dose. When this was not possible, a single sample was taken at no fixed time point following their second vaccine dose. To facilitate sampling, individuals were given the option of remote sampling by dried blood spot (DBS) or for an enhanced cohort venous blood sampling to enable cellular analysis. We have previously recorded excellent concordance between serum and DBS samples using this assay [22]. Serum or dried blood samples [22] were tested for the presence of anti-spike glycoprotein antibodies (The Binding Site, Birmingham, UK). Results are reported as an IgGAM ratio (optical density compared with calibrator) and results > 1.0 are defined as seropositive. The ratio provides a semi-quantitative assessment of the magnitude of the antibody responses [23]. Serum samples were also assessed for neutralising capacity using an in-house live virus neutralisation assay. T cell responses were assessed using the T-SPOT®.COVID assay (Oxford Immunotec, Abingdon, UK), an ELISPOT based IFN-gamma release assays utilising peptide pools derived from the SARS-CoV-2 spike and nucleocapsid proteins; 0–4 spots per well is considered negative, 5–7 spots per cell, borderline and greater than 7 spots per well a positive response. Detailed descriptions of the methods are available in the Supplementary Methods.

### Statistical Analysis

Data were analysed using Graph Pad Prism 9.0 (GraphPad Software, San Diego, California USA). Continuous variables were analysed using the 2-tailed Mann-Whitney *U* test, categorical variables analysed using the *χ*2 test and the relationship between antibody response, time and vaccine received by 2-way ANOVA. Spearman’s rank correlation coefficient was used to assess the relationship between antibody concentrations and neutralisation potential.

### Ethical Approval and Funding

This study was approved by the London — Dulwich Research Ethics Committee (REC reference: 21/LO/0162) and funded by the UK Research and Innovation (MR/W002663/1). Serological responses from healthy individuals are from participants recruited to the COVID-19 convalescent (COCO) immunity study (REC reference 20/HRA/1817). All participants provided written informed consent prior to participation in this study.

## Results

The results of 320 participants in the COV-AD study were available for interim analysis (Table 1). The median age of participants was 58.5 years and 40% (*n* = 128/320) were male. The median interval between the first and second vaccine dose was 76 days; 42.1% (*n* = 135/320) of participants received the Pfizer BioNTech 162b2 vaccine and 55.0% (*n* = 176/320) the AstraZeneca ChAdOx1 nCoV-19 vaccine.

**Table 1:** Demographics of COV-AD study participants

N	320
Age (y)	58.5 (43.0–68.8)
Sex (male) n, %	128 (40.0)
Vaccination	
Pfizer BioNTech 162b2	135 (42.1)
AstraZeneca ChAdOx1 nCoV-19	176 (55.0)*
Unvaccinated	2 (0.63)
Unknown	7 (2.2)
Vaccine dosing interval (d)	76 (70–78)
Diagnosis	
Primary immunodeficiency	228 (71.3)
Common variable immunodeficiency disorder	139 (43.4)
Other primary antibody deficiency	38 (11.8)
Specific polysaccharide antibody deficiency	17 (5.3)
X-linked agammaglobulinemia	9 (2.8)
Hyper IgM syndrome	6 (1.9)
Undefined combined immunodeficiency	4 (1.3)
Thymoma with immunodeficiency	3 (0.9)
Other	12 (3.8)
Secondary immunodeficiency	90 (28.1)
Haematological cause	62 (19.3)
Rheumatological cause	18 (5.6)
Other cause	10 (3.1)
Unknown	2 (0.6)
Immunoglobulin product	
Intravenous immunoglobulin	167 (52.2)
Subcutaneous immunoglobulin	133 (41.6)
Prophylactic antibiotics only	16 (5.0)
Unknown	4 (1.3)
Immunoglobulin level	
Pre-treatment IgG (g/L)	3.65 (1.73–4.92)
Trough IgG (g/L)	9.46 (8.20–11.06)
IgA (g/L)	0.16 (0.05–0.61)
IgM (g/L)	0.3 (0.10–0.68)
SARS-CoV-2 PCR status at study entry	
Positive	1 (0.31)
Negative	283 (88.4)
Unknown	36 (11.3)

^*^One participant in both the AZ and Pfizer vaccine group had only received one vaccine dose during study follow up to 31/10/21. Both unvaccinated participants had previously recovered from prior PCR + SARS-CoV-2 infection

Eighteen participants (*n* = 18/320, 5.6%) had suffered PCR-proven SARS-CoV-2 infection prior to study entry; these participants were significantly younger (52.0 vs. 59.0 years, *p* = 0.02) than individuals who remained SARS-CoV-2 infection-naive (Table 2). Only one participant remained SARS-CoV-2 PCR positive on study entry. Eleven participants (*n* = 11/18, 61.1%) returned negative nasopharyngeal swabs at the time of study entry and six participants declined further investigation. No specific immunological characteristics defined the population with apparent viral clearance: 4 patients had CVID, 2 other primary antibody deficiencies, 1 Good’s syndrome, 1 XLA and 3 secondary immunodeficiencies; 54.4% (*n* = 6/11) made no persistent serological response to infection (measured at study enrollment) and 36.4% (*n* = 4/11) no serological response to subsequent vaccination. T cell responses were assessed in five of the six seronegative individuals by interferon-gamma ELISPOT: 100% (*n* = 5/5, median spots/10^6^ cell = 158) mounted responses against spike peptide pools and 60% (*n* = 3/5, median spots/10^6^ cells = 45) to the nucleocapsid peptide pools demonstrating T cell immunity may compensate for the absence of humoral immunity under some circumstances.

**Table 2:** Comparison of participants in Arm 1 vs Arm 2 of the COV-AD study

	Prior PCR + SARS-CoV-2 infection	No known PCR + SARS-CoV-2 infection	*p*
*N*	18	302	-
Age (y)	52.0 (30.3–61.5)	59.0 (44.0–69.0)	0.02
Sex (male) *n*, %	7 (38.9)	121 (40.3)	NS

**Fig. 1:**
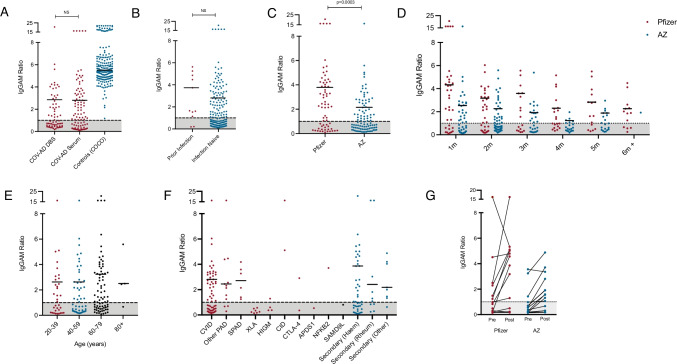
Serological responses to SARS-CoV-2 vaccination in individuals with antibody deficiency. Comparison of serological responses to SARS-CoV-2 vaccination in COV-AD participants. **A** COV-AD participants sampled 1–2 m post second vaccine dose via dried blood spot (DBS) or serum and in comparison to healthy controls (COCO). **B** Comparison of individuals with prior PCR proven infection and those who were infection naive sampled 1–2 m post second vaccine dose. **C** Comparison of recipients of the AstraZeneca ChAdOx1 nCoV-19 and the Pfizer BioNTech 162b2 vaccines sampled 1–2 m post second vaccine dose. **D** Comparison of serological responses over time from vaccination. **E** Comparison of serological responses across age groups in individuals sampled 1–2 m post second vaccine dose. **F** Comparison of serological responses by underlying immunodeficiency in participants samples 1–2 m post second vaccine dose. **G** Dynamic serological response before and after second vaccine dose. Results are presented as the IgGAM ratio with the grey shaded area representing the results below the cut-off for positivity. Horizontal bars represent the median of seropositive results

One hundred and sixty-eight participants were sampled 1 to 2 months after their second vaccine dose using venous or DBS collection. The overall seropositivity following vaccination in this cohort was 54.8% (*n* = 92/168) and the median IgGAM ratio of seropositive individuals was 2.81 (positive defined as ratio > 1.0), with comparable results in groups sampled by DBS and venous blood (Fig. 1A). By comparison, overall seropositivity in 205 healthy participants from the COCO study was 100.0% with a median IgGAM ratio of 5.51. There was no significant difference in the percentage of individuals who were seropositive, or the magnitude of the antibody response between participants who had previously had SARS-CoV-2 infection and those who were infection naive (Fig. 1B). The Pfizer BioNTech 162b2 vaccine was associated with significantly greater seroconversion (65.7% vs. 48.0%, *p* = 0.03) and antibody responses (IgGAM ratio 3.73 vs. 2.39, *p* = 0.0003) in comparison to the AstraZeneca ChAdOx1 nCoV-19 vaccine (Fig. 1C). Serological responses from both vaccines display significant waning over time (2-way ANOVA, *p* = 0.001) but recipients of the Pfizer BioNTech vaccine displayed better preservation of antibody responses (2-way ANOVA, *p* < 0.0001) (Fig. 1D). Age did not significantly affect the magnitude of antibody responses or seroconversion following vaccination (Fig. 1E). Humoral responses following vaccination were variable amongst participants with a range of immunodeficiencies (Fig. 1F). As expected, serological responses were not detected in patients with X-linked agammaglobulinaemia (XLA); however, 52.2% of individuals with common variable immunodeficiency mounted a serological response to vaccination. Seropositivity was 75.0% in individuals with other primary antibody deficiencies (excluding XLA and CVID) and 100.0% in individuals with specific polysaccharide antibody deficiency. Variability was also observed amongst individuals with secondary immunodeficiencies regardless of aetiology. Thirty-one participants were sampled before and after their second immunisation permitting comparison of pre and post vaccine responses (Fig. 1G): seropositivity increased from 29.0% following the first dose to 61.2% following the second dose; both vaccines increased the magnitude of the antibody response.

**Fig. 2:**
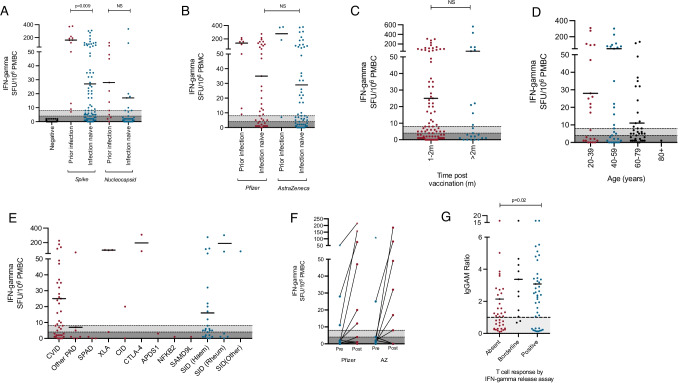
Cellular responses to SARS-CoV-2 vaccination in individuals with antibody deficiency. Comparison of cellular responses following SARS-CoV-2 vaccination in COV-AD participants using an IFN-gamma release assay. **A** Comparison of T cell responses to spike and nucleocapsid peptide pools in individuals with prior PCR proven SARS-CoV-2 infection and those who were infection naive, in participants sampled 1–2 m post second vaccine dose. **B** Comparison of cellular responses to spike peptide pools between the Pfizer and AstraZeneca vaccines in participants sampled 1–2 m post second vaccine dose. **C** Comparison of the cellular responses to spike peptide pools over time. **D** Comparison of the cellular responses to spike peptide pools by age in participants sampled 1–2 m post second vaccine dose. **E** Comparison of the cellular responses to spike peptide pools by underlying immunodeficiency in participants sampled 1–2 m post second vaccine dose. **F** Dynamic changes in cellular response to spike peptide pools before and after the second vaccine dose. **G** Relationship between the T cell response to spike peptide pools and the magnitude of the anti-spike antibody response in participants sampled 1–2 m post second vaccine dose. Results are presented as the number of IFN-gamma producing spots per 10^6^ cells. Dark grey shaded areas represent no response, light grey shaded areas represent borderline response, as per the manufacturers’ instructions

T cell responses to vaccination were studied in 91 infection-naive individuals following their second vaccine dose and 12 individuals with a history of PCR + proven SARS-CoV-2 infection (Fig. 2A). In responses to a peptide pool derived from the SARS-CoV-2 spike protein, 46.2% of infection-naïve participants (*n* = 42/91) mounted a positive T cell response and a further 11.0% (*n* = 10/91) mounted a borderline response. In contrast, 91.7% (*n* = 11/12) of individuals with prior PCR positive infection mounted a positive T cell response to pooled spike peptides and 8.3% (*n* = 1/12) mounted a borderline response, as defined by this assay. In response to the SARS-CoV-2 nucleocapsid peptide pool, 8.8% (*n* = 8/91) of infection-naïve participants demonstrated a detectable T cell response and 1.1% (*n* = 1/91) mounted a borderline response compared to 66.7% (*n* = 8/12) and 8.3% (*n* = 1/12) respectively, in the prior-infection group. Individuals who had suffered previous PCR + SARS-CoV-2 infection mounted a significantly greater post-vaccination T cell response to the spike protein than those who were infection naive; no significant difference was observed for the nucleocapsid protein (Fig. 2A). All eight individuals with no prior history of PCR-proven SARS-CoV-2 infection who had positive T cell responses to nucleocapsid peptides also mounted above average responses to the spike peptide pools (> 100 spot forming units/10^6^ PBMC) suggesting a minority of individuals may have had asymptomatic infection, or mild symptomatic COVID-19 that was incorrectly attributed to other causes.

T cell responses directed towards the spike protein were comparable between the Pfizer BioNTech 162b2 and AstraZeneca ChAdOx1 nCoV-19 following the second dose of either vaccine (Fig. 2B) and persisted as time passed following vaccination (Fig. 2C). Participant age did not significantly influence the percentage of participants mounting a T cell response to the spike protein; a trend was observed towards greater magnitude responses in younger participants (Fig. 2D). A total of 57.9% of participants with common variable immunodeficiency disorder mounted a T cell response to the SARS-CoV-2 spike protein following vaccination with a wide range of responses detected in other primary and secondary immunodeficiencies (Fig. 2E). Both the Pfizer and AstraZeneca vaccines induced incremental T cell responses following the second vaccine doses in the majority of participants (Fig. 2F). A detectable T cell response was associated with significantly greater seropositivity following vaccination (79.5% vs. 53.8%, *p* = 0.009) and antibody responses of significantly greater magnitude (IgGAM ratio 3.08 vs. 2.14, *p* = 0.03) (Fig. 2G); however, no significant relationship was observed between the T cell response and peripheral CD19 + B cell numbers (Supplementary Fig. 2).

**Fig. 3:**
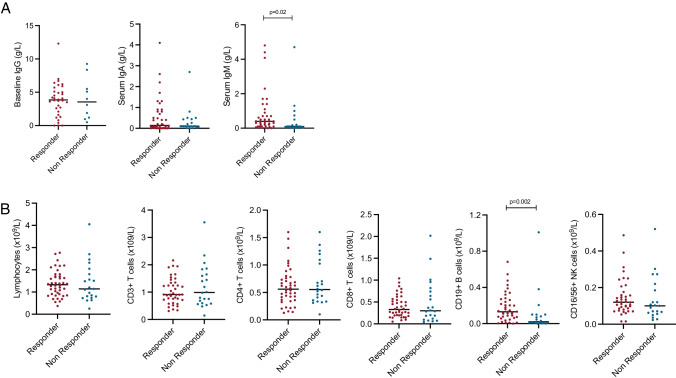
Correlates of seropositivity following SARS-CoV-2 vaccination in individuals with antibody deficiency. Comparison of pre-vaccination immunological parameters between seropositive and seronegative participants sampled 1–2 months following their second vaccine dose: **A** Pre-treatment serum IgG concentration and current serum IgA and IgM concentration. **B** Total lymphocyte count and lymphocyte subset enumeration

Participants that were seropositive post-vaccination had significantly greater serum IgM concentrations (Fig. 3A) and significantly larger numbers of CD19 + peripheral B cells (Fig. 3B) compared to those who were seronegative. There was no direct relationship between CD19 B cell numbers and IgM concentration (*r*^2^ = 0.001, *p* = 0.53). Serum concentrations of both IgA and IgM were positively correlated with the magnitude of the antibody response following vaccination (Supplementary Fig. 1).

**Fig. 4:**
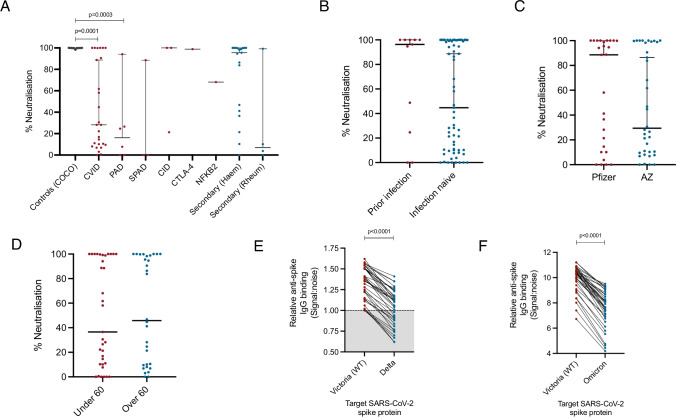
Functional immunity following SARS-CoV-2 vaccination in individuals with antibody deficiency. Serum neutralisation capacity was assessed using live virus neutralisation in seropositive individuals sampled 1–2 months post second vaccine dose. **A** Serum neutralising antibody capacity of seropositive individuals by underlying immunodeficiency. **B** Comparison of serum neutralising antibody capacity between individuals with prior PCR proven SARS-CoV-2 infection and those who were infection naive. **C** Comparison of serum neutralising antibody capacity between recipients of the Pfizer and AstraZeneca vaccinations. **D** Comparison of serum neutralising antibody capacity by age of participants. **E** Comparison of binding of vaccine-induced IgG antibodies from participants sampled 1–2 months post vaccination to the wild-type (Victoria) SARS-CoV-2 spike protein and the Delta variant of concern within an ELISA detection system. **F** Comparison of binding of vaccine-induced IgG antibodies from participants sampled 1–2 months post vaccination to the wild-type (Victoria) SARS-CoV-2 spike protein and the Omicron variant of concern within an ELISA detection system

The functionality of antibodies was studied using in vitro, live virus neutralisation assays. Only 37% of participants with CVID (*p* = 0.0001) and 16% with primary antibody deficiency (*p* = 0.0003) displayed 50% viral neutralising activity or greater, compared to 100% of healthy controls (Fig. 4A). Neutralising capacity was not significantly impacted by prior SARS-CoV-2 infection status (Fig. 4B), type of vaccination received (Fig. 4C) or by participants’ age (Fig. 4D). The capacity of vaccine induced anti-spike IgG antibodies to bind the SARS-CoV-2 delta variant was significantly reduced compared to original Victoria strain (Normalised signal:noise ratio: 1.26 vs. 1.41, *p* < 0.0001). A total of 39.4% of individuals with detectable IgG responses against the Victoria strain fell below the threshold for positivity when the delta variant was substituted into the ELISA assay (Fig. 4E). Vaccine-induced IgG antibody binding was also significantly reduced to the Omicron variant of concern compared to original virus (Normalised signal:noise ratio 7.66 vs. 10.32, *p* < 0.0001) (Fig. 4F); however, no participants fell below the threshold for positivity.

Ten vaccine-breakthrough, PCR-proven infections have occurred this cohort up to 31/10/21 (median time from 2nd vaccine dose: 197 day); a further individual was infected between their first and second vaccine dose on the background of prior COVID-19 (Table 3). Eight participants reported new symptoms associated with acute COVID-19 above and beyond any chronic symptoms secondary to their immunodeficiency. A total of 90.0% (*n* = 9/10) of vaccine-breakthrough infections occurred in recipients of the AstraZeneca vaccine at a median interval of 120 days post second-dose and 70.0% occurred in individual who made no detectable humoral response to vaccination. One participant died of COVID-19, 3 months after receiving their second vaccine dose. This participant had a 31-year history of seropositive rheumatoid arthritis and secondary antibody deficiency (nadir IgG prior to immunoglobulin replacement: 0.97 g/L). Prior treatments for the underlying rheumatoid arthritis included oral corticosteroids, methotrexate, hydroxychloroquine and abatacept. At the time of SARS-CoV-2 infection, this participant was receiving daily oral prednisolone (9.5 mg/day) and had received rituximab 84 days prior to their first vaccine dose and 41 days after their second vaccine dose. No serological or cellular response to vaccination was detected in this participant at study enrolment.

**Table 3:** Vaccine breakthrough infections in COV-AD participants

Patient	Age	Sex	Diagnosis	Prior COVID	Symptoms and severity	Duration of positivity (*d*)	Time from second vaccine dose to infection (*d*)	Vaccine	Seropositive post vaccination	IgGAM ratio	B cell count (× 10^9^/L)	Outcome
1	54	F	Undefined CID	Y	Y – mild, non-hospitalised, no specific treatment	Not known -declined swabs	1 dose only	AZ	Y	5.41	4.59	Alive
2	68	F	Secondary—rheumatology	N	Y – hospitalised, COVID pneumonia treated with dexamethasone and remdesivir	29	94	AZ	N	0.25	Undetectable	Deceased
3	38	M	CVID	N	Y – mild, non-hospitalised, no specific treatment	28	116	AZ	Y	2.47	0.17	Alive
4	38	F	Primary antibody deficiency	N	Y – mild, non-hospitalised, antibiotics for secondary infection	22	143	AZ	N	0.63	n/a	Alive
5	37	F	CVID	N	Y – mild, non-hospitalised, antibiotics for secondary infection	Not known -declined swabs	138	AZ	N	0.27	0.01	Alive
6	60	M	CVID	N	Unknown	80^*^	92	Pfizer	N	0.19	0.03	Alive
7	61	F	Secondary—haematological	N	Unknown	11^$^	181	AZ	Y	1.59	0.13	Alive
8	45	F	CVID	N	Y – mild, non-hospitalised, antibiotics for secondary infection	63	61	AZ	N	0.18	0.01	Alive
9	44	M	CVID	N	Y – mild, non-hospitalised, no specific treatment	16	111	AZ	N	0.48	0.01	Alive
10	50	F	Secondary—haematological	N	Y – mild, non-hospitalised, antibiotics for secondary infection	24^$^	199	AZ	N	0.62	0.45	Alive
11	61	M	CVID	N	Unknown	10	120	AZ	Y	2.26	0.24	Alive

^*^Received Casirivimab/imdevimab prior to viral clearance. ^$^Ongoing infection at time of publication. Acronyms: *Y* Yes, *N* No, *AZ* AstraZeneca ChAdOx1 nCoV-19, *CID* combined immunodeficiency disorder, *CVID* common variable immunodeficiency disorder, *Pfizer* Pfizer BioNTech 162b

## Discussion

Understanding the immunogenicity and efficacy of vaccinations is essential to guide global vaccination strategies and when to deploy non-pharmacological countermeasures to protect the immunologically vulnerable [19, 20]. Herein, we report the immunogenicity of the AstraZenca ChAdOx1 nCoV-19 and Pfizer BioNTech 162b2 vaccinations in patients with antibody deficiency, a cohort who have historically responded poorly to vaccinations [24–26].

Overall, seropositivity following vaccination was 54.8%, significantly lower than healthy controls; comparable seropositivity was observed in the two largest subgroups of patients, common variable immunodeficiency (52.1%) and secondary immunodeficiency arising from haematological cause (55.8%). However, less than 10% of individuals with primary or secondary antibody deficiency made a neutralising antibody response equivalent to that of healthy controls following two doses of a SARS-CoV-2 vaccine. Furthermore, in individuals demonstrating a vaccine response, anti-spike IgG binding was significantly reduced against both the Delta and Omicron SARS-CoV-2 variants of concerns which are in widespread global circulation as of December 2021. Evidence suggests antibody binding is strongly associated with neutralising capacity [27]. These data suggest that vaccine-induced antibody responses are inadequate in the majority of individuals with antibody deficiency and additional strategies such as the use of prophylactic monoclonal antibodies to provide passive protection and antivirals are likely to be necessary to prevent severe disease.

T cell responses to vaccination displayed significant heterogeneity in this cohort as has been found in similar studies using identical laboratory methods [28]. The interferon-gamma release assay was originally validated to study T cell responses following natural infection, where it displays 98% sensitivity [29]. A total of 46.2% of infection-naïve COVAD participants and 91.7% of individuals with prior PCR-proven infection mounted a detectable T cell response following vaccination using this assay, compared to 54% of healthy individuals [30]. T cell responses in patients with evidence of previous SARS-CoV-2 infection were significantly greater than those detected following vaccination in infection-naive participants. The discordance between the detection of vaccine- and infection-induced T cell responses may arise from the duration, anatomy and magnitude of antigen exposure, differences in the immunological environment when antigen was presented, or assay-specific factors including differences in MHC restriction to the constituents of the peptide pool and the antigen-specific T cell repertoire in each circumstance.

Strong, polyfunctional T cell responses have previously been shown in an XLA patient following infection [5], and concordant with our study results, T cell response have been demonstrated in the majority of XLA patients following vaccination [17, 18]. However, across our antibody deficient cohort, there were no differences between the magnitude of the T cell response in individuals with or without a detectable peripheral B cell population. The clinical correlates of infection- or vaccine-induced T cell responses in patients with antibody deficiency, in particular, in the absence of humoral immunity remain uncertain. The absence of humoral immunity is a characteristic feature of individuals with prolonged SARS-CoV-2 infection [7]; however, robust T cell responses can limit the severity of disease in some individuals in the absence of humoral immunity as has been shown previously in patients with haematological malignancy [31]. Further studies are necessary to characterise the quality and breadth of T cell responses and its relationship to the development of effective humoral immunity following infection and vaccination in more detail.

With respect to vaccination strategies, we have shown that the Pfizer BioNTech 162b2 vaccine demonstrated significantly greater humoral immunogenicity in patients with antibody deficiency than the AstraZeneca ChAdOx1 nCoV-19 vaccination, a finding consistent with larger studies in healthy individuals [13, 32] and renal transplant recipients [28]. Furthermore, over 90% of vaccine breakthrough infections occurred in recipients of the AstraZeneca vaccine, 60.0% of whom made no serological response to the initial 2-dose vaccine schedule. Studies in the general population have suggested adenoviral-vectored vaccines demonstrate reduced vaccine-efficacy against severe disease when directly compared to mRNA vaccines (https://www.cdc.gov/mmwr/volumes/70/wr/mm7038e1.htm). These observations support the use of mRNA vaccines in patients with antibody deficiency.

It could be argued that the deployment of a 3rd dose of vaccination in individuals that have not responded to a first dose is futile. However, we have found that serological and cellular responses to the SARS-CoV-2 spike protein were positively incremented by the second vaccine dose, in keeping with previous studies in patients with inborn errors of immunity [16] suggesting potential benefit from further doses. Our group have demonstrated the effectiveness of a 3rd primary immunisation in raising antibody levels against the delta and omicron variants of concern in a cohort of immunocompromised renal dialysis patients and provide preliminary evidence of the benefit of a heterologous vaccination strategy on serological responses to vaccination [33]. Further studies will be necessary to explore whether different vaccination combinations (homologous/heterologous) or dosing schedules may improve responses and efficacy in patients with primary and secondary humoral immunodeficiencies.

Existing studies have reported wide variation in the serological response to vaccination in patients with immunodeficiency: post-vaccine seroprevalence have ranged from 20.0 to 80.0% [14–18]. The COVAD study is the largest reported study of patients with antibody deficiency and finds a seropositivity rate of 54.8% overall. At a cohort level, total B cell numbers were the principal determinate of serological response to vaccination, also in keeping with other SARS-CoV-2 vaccine studies [17]. Additional correlates of vaccine responsiveness remain to be elucidated: Salinas et al. demonstrated patients with CVID have a relative paucity of receptor-binding domain-specific, CD19^+^ CD24^+^ CD27^+^ B cells compared to healthy controls [18] and Hagin et al. were unable to demonstrate a common T-cell immunophenotype in vaccine non-responders beyond an inverted CD4/CD8 ratio [17]. Future work in COV-AD will employ detailed phenotypic and functional profiling to investigate potential correlates of vaccine immunogenicity and efficacy within this heterogeneous cohort.

This is a large study in a rare disease cohort and, although heterogeneous, we have had the opportunity to compare the immunogenicity of an mRNA and adenoviral-vectored vaccine in an immunodeficient cohort. To some extent, the generalisability of our study to the wider world is confounded by the extended UK vaccine schedule, which has not yet been widely adopted elsewhere. On the one hand, extension of the interval between first and second doses has been associated with greater neutralising antibody responses and enrichment of virus specific CD4 + T cells in healthy individuals [34], but shorter dosing intervals were associated with better humoral responses in a smaller study of patients vaccinated following treatment with B cell depleting agents [35]. There is an urgent need for further studies that explore how to maximise vaccine immunogenicity and efficacy in larger and heterogeneous cohorts of immune deficient patients.

In conclusion, we demonstrate profound impairment of serological responses following SARS-CoV-2 vaccination in patients with antibody deficiency and evidence of the superior immunogenicity of the Pfizer BioNTech 162b2 vaccine. These data highlight the ongoing risk of SARS-CoV-2 infection in antibody deficiency patients and should inform public health policy on vaccination strategies and other treatments to prevent morbidity and mortality.

## Supplementary Information

Below is the link to the electronic supplementary material.

Supplementary file1 (DOCX 22 KB)

Supplementary file2 (PDF 22 KB)

## Data Availability

The datasets generated during and/or analysed during the current study are available from the corresponding author on reasonable request.

## Abbreviations

APDS-1: Activated PI3K delta syndrome 1
CID: Combined immunodeficiency
COVID-19: Coronavirus disease 2019
CTLA-4: Cytotoxic T-lymphocyte-associated protein 4
CVID: Common variable immunodeficiency disorder
DBS: Dried blood spot
ELISA: Enzyme-linked immunosorbent assay
NFKB2: Nuclear factor kappa B subunit 2
PAD: Primary antibody deficiency
PCR: Polymerase chain reaction
SAMD9L: Sterile alpha motif domain containing 9 like
SARS-CoV-2: Severe acute respiratory syndrome coronavirus 2
SID: Secondary immunodeficiency
SPAD: Specific polysaccharide antibody deficiency
XLA: X-linked agammaglobulinaemia
