# Supplementary material for: SARS-CoV-2 Vaccine Responses in Individuals with Antibody Deficiency: Findings from the COV-AD Study
Source: J Clin Immunol. 2022 Apr 14;42(5):923–34. doi: 10.1007/s10875-022-01231-7 (PMC9008380; doi:10.1007/s10875-022-01231-7)
Supplement: Supplementary file 1 — Supplementary file1 (DOCX 22 KB) [file 10875_2022_1231_MOESM1_ESM.docx]

# Supplementary Methods

*SARS-CoV-2 antibody testing*

Participants were offered the opportunity to participate via venous blood sampling requiring hospital visits, or remotely via analysis of self-collected dried blood spots (DBS). Serological responses to SARS-CoV-2 vaccination were determined using and anti-IgG/A/M SARS-CoV-2 ELISA (The Binding Site, Birmingham, UK), an assay previously validated to have a sensitivity and specificity of 98.6% and 98.3% respectively [1]. This method was used to analyse both serum and DBS samples; these methods have previously demonstrated a high degree of concordance [2]. Any result greater than 1.0 is considered seropositive; results are reported as the percentage of participants who are seropositive and the median of the IgGAM ratio in seropositive participants.

*Neutralisation studies*

Vero cells were seeded in 96-well plates in Dulbecco’s Modified Eagles Medium with 10% FBS, 1% penicillin-streptomycin, 1% L-Glutamine. SARS-CoV-2 virus (10^6^ IU/ml stock, PHE SARS-CoV-2 England 2) at final dilution 1/300 was incubated with the patient sample sera for 1 hour at 37^o^C and added to Vero cells for 48 hours. Serum dilutions of both 1/50 and 1/200 were tested for neutralising ability with a 1/200 dilution showing a stronger relationship (r=0.47, p<0.0001) with serum antibody concentration (**Supplementary Figure 3**). The cells were fixed with methanol for 5 minutes and stained with rabbit anti-SARS-CoV-2 clone CR3022 (Native Antigen Company, Oxford, UK) and anti-rabbit Alexaflour 555 (Thermo Fisher Scientific) and Hoechst 33420 nuclear staining. The plates were imaged using a Thermo CellInsight CX5 high content screening platform and custom algorithms were used to quantify percentage infection and number of viable cells. Results are expressed as percentage neutralisation with reference to antibody negative control sera. Only serum that had tested positive for anti-SARS-CoV-2 anti-spike antibodies by ELISA were evaluated using neutralising assays

*Comparative binding of antibodies to SARS-CoV-2 variant of concern spike proteins*

Wuhan and Delta spike variant produced in a 293 protein expression system were kindly obtained from Abingdon Health. His-tagged Wuhan and Omicron spike proteins were obtained from Sino Biological. All spike proteins were coated at 1 ug/mL (50 ul) onto high binding ELISA plates overnight at 4 degrees C. Plates were washed with PBS-Tween20 and then blocked with 2% BSA diluted in PBS Tween (0.1% Tween-20) for 1 hour at room temperature (RT). Serum samples were diluted at 1:40 with 2% BSA + 0.1% PBS-Tween and added to the plate (100 ul) after washing and incubated for 1 hour at RT. HRP-conjugated mouse-anti-human R10-IgG (monoclonal antibody obtained from Dr. Margaret Goodall) (1:8000 dilution, 100 ul) was then added to the plate and incubated for 1 hour at RT. Plates were washed and then TMB substrate was used for development. 0.2M H_2_SO_4_ (50 ul) was added to the well after 10 minutes in order to stop the reaction. Optical densities were measured at 450 nm on the Dynex Dynaread (Aspect Scientific). The cutoff for positivity for each assay was determined using pre-pandemic control sera (n=47 Wuhan, n=74 Omicron) with the OD cutoff set at the mean OD + 3sd of the mean OD in each case. For comparison, results from patient samples have been normalized to the cutoff. Direct comparison of IgG binding to Delta and Omicron binding is not possible at the time of these experiments due to the different manufacture and purification of the viral spike proteins from Abingdon Health and Sino Biological.

*SARS-CoV-2 interferon gamma release assay*

IFN-gamma release assays were performed using the T-spot.COVID assay as per the manufacturers instructions (Oxford Immunotec, Abingdon, UK). Briefly, peripheral blood mononuclear cells (PBMCs) are stimulated with proprietary peptide pools derived from the spike or nucleocapsid proteins of the SARS-CoV-2 virus for 16-20 hours alongside negative and positive controls. IFN-gamma producing colonies are enumerated by ELISPOT: 0-4 spots per well is considered negative, 5-7 spots per cell, borderline and greater than 7 spots per well a positive response.

# References

1. Cook, A.M., et al., Validation of a combined ELISA to detect IgG, IgA and IgM antibody responses to SARS-CoV-2 in mild or moderate non-hospitalised patients. J Immunol Methods, 2021. **494**: p. 113046.

2. Morley, G.L., et al., Sensitive Detection of SARS-CoV-2-Specific Antibodies in Dried Blood Spot Samples. Emerg Infect Dis, 2020. **26**(12): p. 2970-2973.
