# Supplementary material for: SARS-CoV-2 Vaccine Responses in Individuals with Antibody Deficiency: Findings from the COV-AD Study
Source: J Clin Immunol. 2022 Apr 14;42(5):923–34. doi: 10.1007/s10875-022-01231-7 (PMC9008380; doi:10.1007/s10875-022-01231-7)
Supplement: Supplementary file 2 — Supplementary file2 (PDF 22 KB) [file 10875_2022_1231_MOESM2_ESM.pdf]

Supplementary Figure 1: Correlates of magnitude of vaccine response

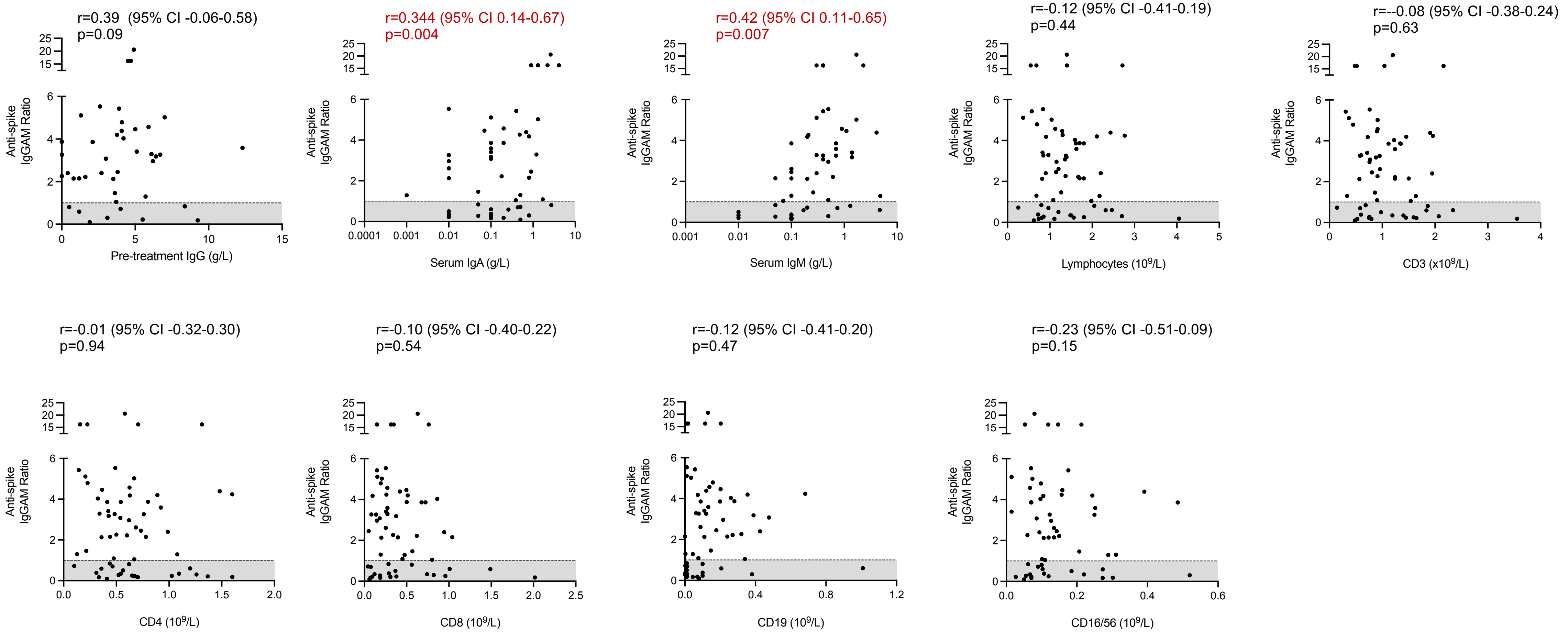

Supplementary Figure 2: Relationship between B cell numbers and post-vaccine T cell responses

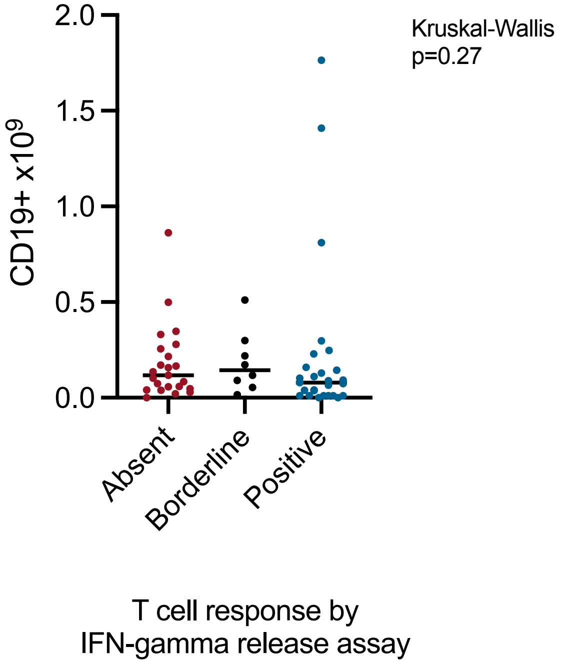

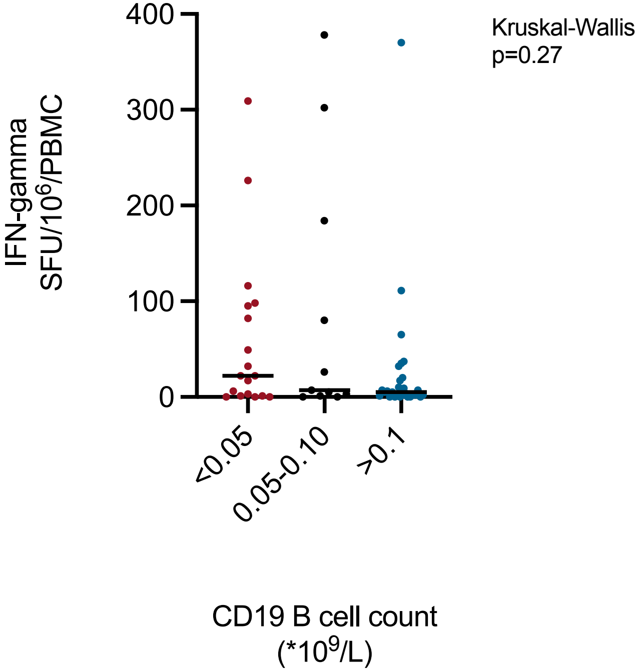

Supplementary Figure 3: Correlation between IgGAM levels and neutralization response

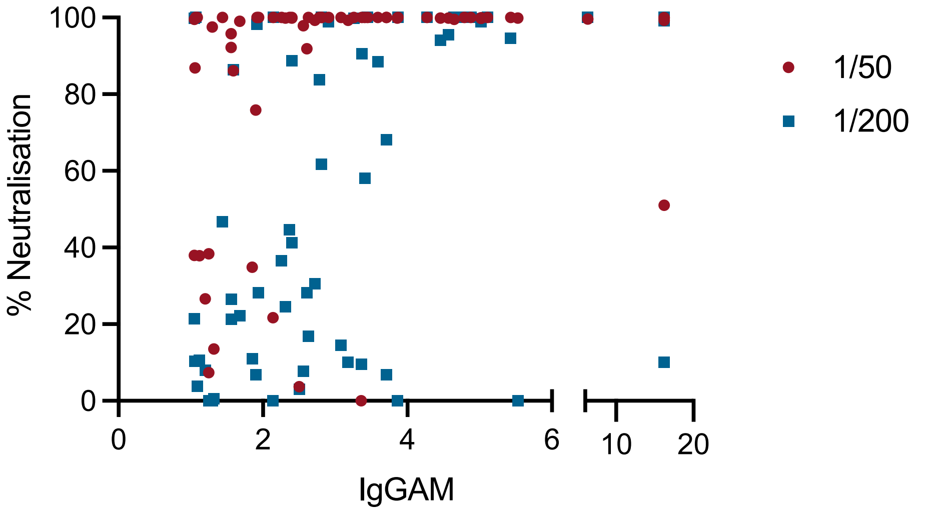

1/50 – r=0.34, 95% CI 0.10-0.55, p=0.059

1/200 – r=0.47, 95% CI = 0.25-0.65, <0.0001
